# Temporal Stability of Genetic Structure in a Mesopelagic Copepod

**DOI:** 10.1371/journal.pone.0136087

**Published:** 2015-08-24

**Authors:** Erica Goetze, Kimberly R. Andrews, Katja T. C. A. Peijnenburg, Elan Portner, Emily L. Norton

**Affiliations:** 1 Department of Oceanography, School of Ocean and Earth Science and Technology, University of Hawaii at Manoa, Honolulu, Hawaii, 96822, United States of America; 2 Department of Fish & Wildlife Sciences, University of Idaho, Moscow, Idaho, 83844, United States of America; 3 Naturalis Biodiversity Center, P.O. Box 9517, 2300 RA, Leiden, The Netherlands; 4 Institute for Biodiversity and Ecosystem Dynamics (IBED), University of Amsterdam, P.O. box 94248, 1090 GE, Amsterdam, The Netherlands; University of Connecticut, UNITED STATES

## Abstract

Although stochasticity in oceanographic conditions is known to be an important driver of temporal genetic change in many marine species, little is known about whether genetically distinct plankton populations can persist in open ocean habitats. A prior study demonstrated significant population genetic structure among oceanic gyres in the mesopelagic copepod *Haloptilus longicornis* in both the Atlantic and Pacific Oceans, and we hypothesized that populations within each gyre represent distinct gene pools that persist over time. We tested this expectation through basin-scale sampling across the Atlantic Ocean in 2010 and 2012. Using both mitochondrial (mtCOII) and microsatellite markers (7 loci), we show that the genetic composition of populations was stable across two years in both the northern and southern subtropical gyres. Genetic variation in this species was partitioned among ocean gyres (*F*_*CT*_ = 0.285, *P* < 0.0001 for mtCOII, *F*_*CT*_ = 0.013, *P* < 0.0001 for microsatellites), suggesting strong spatial population structure, but no significant partitioning was found among sampling years. This temporal persistence of population structure across a large geographic scale was coupled with chaotic genetic patchiness at smaller spatial scales, but the magnitude of genetic differentiation was an order of magnitude lower at these smaller scales. Our results demonstrate that genetically distinct plankton populations persist over time in highly-dispersive open ocean habitats, and this is the first study to rigorously test for temporal stability of large scale population structure in the plankton.

## Introduction

Understanding the ecology and evolution of marine species requires knowledge of the extent to which conspecific populations are genetically differentiated across space, and whether these spatial patterns are stable through time. At large geographic scales, marine species with large and stable population sizes, broad species ranges, long-lived planktonic larvae, and high fecundity are expected to exhibit temporal stability in allele frequencies coupled with weak or no genetic structure among populations (e.g., [1]), due to the combined effects of low genetic drift and high migration among sites. Although the expectation of weak population differentiation in such ‘high gene flow’ species is commonly observed (e.g., [2–6]), temporal stability in allele frequencies has been documented in very few cases [7,8]. In contrast, there is an extensive literature describing unexpectedly high levels of non-geographic differentiation among samples (e.g., [9–11]), termed chaotic genetic patchiness [12,13], due to genetic variation at fine temporal and/or spatial scales. In benthic species with meroplanktonic larvae, this fine-scale pattern appears to be largely driven by high variance in reproductive success, coupled with stochasticity in larval survivorship and transport due to variation in oceanographic conditions (e.g., [14–16]). Similar patterns also have been reported for pelagic marine fish. For example, in the European eel, results previously considered to represent spatial structuring among populations have since been suggested to reflect temporal heterogeneity [17,18], with both isolation by spawning cohorts as well as variance in reproductive success within cohorts contributing to differentiation among samples.

Marine holozooplankton, ocean drifters during their entire lifecycle, arguably represent the end of a continuum for marine animals: They are characterized by exceptionally large population size and among the greatest dispersal potential of all marine species. Because of these characteristics, we expect to observe little or no genetic differentiation among populations, and high temporal stability in allele frequencies at large spatial scales. However, despite their very high capacity for dispersal in the open sea, many holozooplankton species are strongly differentiated among populations at ocean gyre and basin spatial scales [19]. For example, populations in the northern and southern subtropical gyres of the Atlantic, Pacific and Indian Oceans exhibit strong genetic differentiation in the cosmopolitan copepods *Eucalanus spinifer*, *Eucalanus hyalinus*, and *Pleuromamma xiphias* [20,21], and the temperate-boreal North Atlantic species *Meganyctiphanes norwegica* (euphausid) and *Sagitta setosa* (chaetognath) also have genetically distinct populations across water masses and/or across European coastal seas [22–25]. Yet virtually nothing is known regarding the stability of spatial genetic structure in marine zooplankton, because temporally-replicated sampling remains very rare. Important exceptions include work on two krill species [25,26] and a chaetognath [27], in which temporally-replicated samples in certain areas showed that the genetic composition was stable over time. However, since sampling in these studies was not replicated over the entire spatial scale of study, it has never been explicitly tested whether the pattern of spatial structuring was temporally stable.

In this study, we examine the temporal stability of spatial population genetic structure across oceanic habitats in the mesopelagic copepod *Haloptilus longicornis*. This common upper-mesopelagic species has been shown in prior work to have genetically distinct populations within subtropical gyres of both the North and South Pacific and the North and South Atlantic Oceans [28,29]. Spatial patterns of abundance in the Atlantic Ocean for this species indicate that subtropical gyres are the preferred habitat, and that although the species is present in equatorial waters, this region serves as a biophysical barrier to migration among gyre populations [30]. Previous analyses based on both mtDNA and nuclear microsatellite markers indicate that the nominal species *H*. *longicornis* is composed of two morphologically cryptic species [28]. Throughout the remainder of the paper we refer to these as *H*. *longicornis* species 1 and *H*. *longicornis* species 2 (or sp. 1 & sp. 2). Both species are circumglobal in distribution, and occur sympatrically across subtropical and tropical waters. This study focuses on understanding temporal genetic patterns within *H*. *longicornis* species 1, the more abundant of the two species.

Given prior observations of significant population structure in *H*. *longicornis*, this system presents an opportunity to test whether genetically distinct populations of marine zooplankton persist in open ocean habitats. In this study, we addressed the questions: (1) Are populations of *H*. *longicornis* species 1 sampled in the northern and southern subtropical gyres of the Atlantic Ocean in 2010 genetically indistinguishable from populations found in these same ocean regions in 2012? and (2) Is the spatial population structure observed across ocean gyres temporally stable, and present during both sampling years? To answer these questions, we used material from repeat basin-scale transects in the Atlantic Ocean, and data from both mitochondrial and microsatellite markers to assess population differentiation across time and space in this system. This study is the first to rigorously test for temporal stability of population structure in oceanic zooplankton.

## Materials and Methods

### Plankton collections and oceanographic data

**Fig 1:**
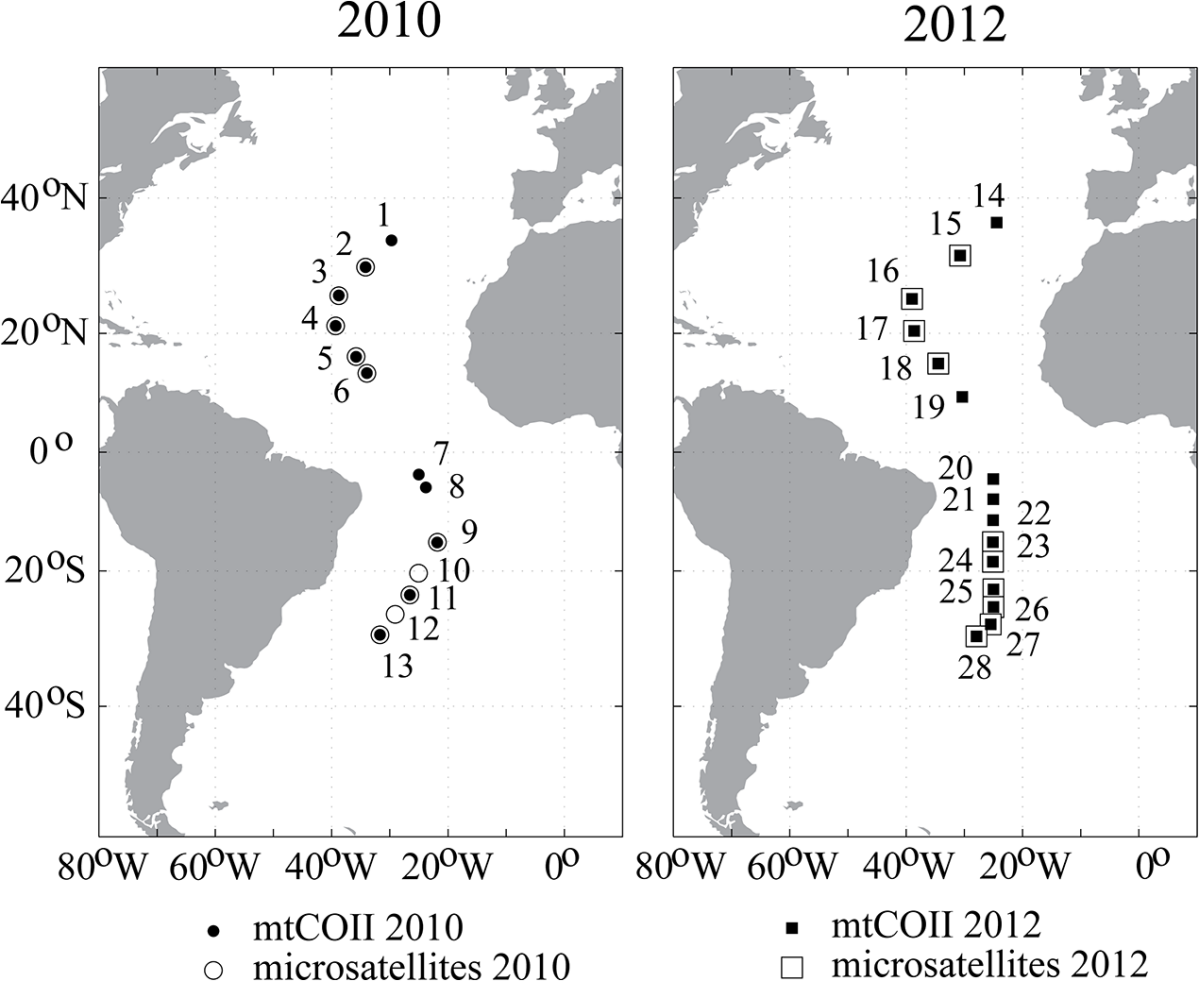
Map of collection locations from the 2010 and 2012 Atlantic Meridional Transect (AMT) cruises that were included in this study. Symbols for sites included in mtDNA and microsatellite analyses as shown in the legend (and as listed in Table 1).

Bulk plankton samples were collected on the Atlantic Meridional Transect (AMT) cruises in 2010 and 2012 (AMT20, AMT22; Fig 1, Table 1). Permits were not required for these collections, and the work did not involve endangered or protected species. On the 2010 cruise, plankton collections were made by vertical casts of a 0.57-m diameter bongo net (200 μm, 200–0 m depths). On the 2012 cruise, a 0.71-m diameter bongo net (200 μm mesh) was towed obliquely between on average 336 m and the surface. This study includes material from 12 stations on the 2010 cruise and 15 stations on the 2012 cruise (Fig 1, Table 1), with collection locations and dates as listed in Table 1. Bulk plankton was preserved immediately in 100% ethyl alcohol, alcohol was changed within 24 hours of collection, and samples were stored at -20°C. Adult females of *Haloptilus longicornis* were identified to species following Bradford-Grieve [31]. Note that *H*. *longicornis* species 1 and *H*. *longicornis* species 2 cannot yet be distinguished morphologically, and both species were included in DNA sequencing and microsatellite genotyping (Table 1). DNA was extracted from individual specimens using the DNeasy Blood & Tissue Kit (QIAGEN), with modifications to the manufacturer’s protocol as described in Norton and Goetze [29].

**Table 1:** Collection sites for specimens of *Haloptilus longicornis* s.l. included in this study, from (A) the 2010 AMT cruise (AMT20), and (B) the 2012 AMT cruise (AMT22).

Pop	Station	mtDNA	mtDNA	mtDNA	msat	msat	msat	Latitude	Longitude	Collection
ID		N	sp. 1	sp. 2	N	sp. 1	sp. 2			Date
*(A) 2010 Cruise*, *Atlantic Meridional Transect Cruise 20*										
1	AMT 20–07	28	11	17	-	-	-	34°12.19'N	29°43.31'W	10/21/10
2	AMT 20–09	31	28	3	43	37	6	30°17.29'N	34°10.86'W	10/23/10
3	AMT 20–11	27	27	0	44	43	1	25°59.10'N	38°46.98'W	10/25/10
4	AMT 20–13	26	26	0	45	45	0	21°12.71'N	39°17.59'W	10/27/10
5	AMT 20–15	26	21	5	45	37	8	16°11.42'N	35°48.36'W	10/29/10
6	AMT 20–16	26	19	7	42	30	12	13°27.28'N	38°57.02'W	10/30/10
7	AMT 20–21	25	2	23	-	-	-	3°51.95'S	25°01.06'W	11/5/10
8	AMT 20–22	25	6	19	-	-	-	06°03.44'S	23°48.77'W	11/6/10
9	AMT 20–24	25	21	4	45	37	8	15°19.88'S	21°50.47'W	11/11/10
10	AMT 20–25	-	-	-	45	41	4	20°22.78'S	25°05.35'W	11/13/10
11	AMT 20–26	27	24	3	44	37	7	23°50.27'S	26°34.02'W	11/14/10
12	AMT 20–27	-	-	-	48	32	16	26°51.44'S	29°04.09'W	11/15/10
13	AMT 20–28	26	14	12	44	28	16	29°56.59'S	31°41.40'W	11/16/10
	**Total**	**292**	**199**	**91**	**445**	**367**	**78**			
*(B) 2012 Cruise*, *Atlantic Meridional Transect Cruise 22*										
14	AMT22-11	27	5	22	-	-	-	36°40.36'N	24°26.83'W	10/17/12
15	AMT22-15	28	24	4	57	47	10	32°0.04'N	30°44.21'W	10/19/12
16	AMT22-21	28	24	4	63	60	3	25°28.57'N	39°0.01'W	10/22/12
17	AMT22-25	29	28	1	47	45	2	20°23.96'N	38°36.69'W	10/24/12
18	AMT22-29	26	21	4	68	50	18	15°3.38'N	34°28.45'W	10/26/12
19	AMT22-33	10	0	10	-	-	-	9°27.51'N	30°21.25'W	10/28/12
20	AMT22-43	7	0	7	-	-	-	4°37.29'S	25°1.39'W	11/2/12
21	AMT22-45	27	16	11	-	-	-	8°4.63'S	25°2.39'W	11/3/12
22	AMT22-47	29	18	10	-	-	-	11°36.92'S	25°2.74'W	11/4/12
23	AMT22-49	27	21	6	74	51	23	15°17.99'S	25°4.48'W	11/5/12
24	AMT22-51	27	25	2	49	40	9	18°29.57'S	25°6.03'W	11/6/12
25	AMT22-55	30	24	6	72	52	20	22°56.97'S	25°0.17'W	11/9/12
26	AMT22-57	26	23	3	52	40	12	25°43.65'S	24°59.94'W	11/10/12
27	AMT22-58	29	18	11	82	42	40	28°21.70'S	25°27.27'W	11/11/12
28	AMT22-60	27	26	1	49	46	3	30°10.46'S	27°54.36'W	11/12/12
	**Total**	**377**	**273**	**102**	**613**	**473**	**140**			

Pop ID = the population identifier referred to throughout the manuscript; Station = the cruise and station number of each sample; mtDNA N and msat N are the number of adult females from which mtCOII DNA sequence and microsatellite genotypes were obtained. Total N for each marker type is also separated into *H*. *longicornis* species 1 (mtDNA sp. 1, msat sp. 1) and *H*. *longicornis* species 2 (mtDNA sp. 2, msat sp. 2) at each site.

### MtDNA marker and sequence analysis

A 546 bp fragment of the mitochondrial gene cytochrome oxidase subunit II (mtCOII) was amplified in polymerase chain reaction (PCR) using primers COII_F6 (5’–GTC TAC AGG ATG CAA ACT CC – 3’) and COII_R9 (5’–AGA GCA TTG CCC AAA CCT GA – 3’; [29]). Amplification conditions and preparation of PCR products for sequencing were as described in Norton and Goetze [29], with Sanger sequencing of both forward and reverse strands. Forward and reverse sequences were aligned (Geneious v5.5.3), checked for errors, and unique haplotypes were identified using FaBox v1.41. MtCOII sequences were obtained from 292 and 377 animals collected in 2010 and 2012, respectively (Table 1). Of these, a total of 199 and 273 were of *H*. *longicornis* species 1 (Table 1). Animals were identified to *H*. *longicornis* species 1 based on placement in mitochondrial lineage 1 or into Microsatellite Cluster 1 by Factorial Correspondence Analysis (FCA, *see below*), with complete concordance observed between these two approaches in the placement of animals to species (as described in [28]). All mtCOII sequences from 2012 are first reported on here; data from 2010 were included in [29] and [28]. A sequence alignment was created including only individuals from *H*. *longicornis* species 1 (using MUSCLE, within Geneious v7.1.7, [32]. A phylogenetic tree for all haplotypes was inferred under maximum likelihood (ML) using MEGA v6.06 [33], and the Tamura and Nei (+ G) substitution model. This ML tree was converted to a haplotype genealogy and plotted in Haploviewer (http://www.cibiv.at/~greg/haploviewer).

### Microsatellite markers and genotyping

Individuals were genotyped at 7 microsatellite markers that were developed for *H*. *longicornis* [28,34]: HALOMS027, HALOMS032, HALOMS064, HALOMS066, HALOMS086, HALOMS091, and HALOMS175. Primer sequences and PCR conditions were as reported in [28]. Microsatellite loci were amplified in 10 μl multiplex PCRs containing 1X Type-it Multiplex PCR Master Mix (Qiagen) and 2 μM of each primer. PCR products were genotyped using an ABI3730 Genetic Analyzer, and scoring of microsatellite chromatograms was conducted using GENEMAPPER v4.0. A total of 1058 individuals were genotyped (Table 1). Specimens were assigned to *H*. *longicornis* species 1 or *H*. *longicornis* species 2 based on factorial correspondence analysis (FCA) using GENETIX v4.05 [35], as described in [28]. A total of 840 individuals were assigned to *H*. *longicornis* species 1, and were included in all subsequent analyses (Table 1). From 28 to 60 individuals of *H*. *longicornis* species 1 were genotyped from each site, with medians of 37 and 47 individuals in 2010 and 2012, respectively. All data from 2012 are first reported here; data from 2010 were included in [28].

### Population genetic analyses

For *H*. *longicorni*s species 1, deviations from Hardy-Weinberg equilibrium (HWE) and linkage disequilibrium were examined using ARLEQUIN v3.5.1.3 and GENEPOP v4.2 for all microsatellite loci [36–38]. We tested for the presence of null alleles in microsatellite data using MICROCHECKER v2.2.3 [39], and estimated null allele frequencies and calculated population pairwise *F*_*ST*_ values with correction for null alleles in FreeNA [40]. Microsatellite genetic diversity indices of observed and expected heterozygosity, average alleles per locus, and allele richness were calculated in GENETIX v4.05 and FSTAT [35,41]. Pairwise *F*_*ST*_ values were calculated among all sample sites using both microsatellite and mtCOII data, as a measure of population subdivision across samples (ARLEQUIN v3.5.1.3, [38]). Significance was assessed following correction for multiple comparisons using the false discovery rate (FDR, [42,43]). Pairwise Φ_*ST*_ values also were calculated for the mtCOII data. We identified the nucleotide substitution model that best fit our mtCOII data using the Akaike Information Criterion, as implemented in jModelTest v2.1.4 [44], and the K81 or three-parameter model was selected as the best model (TPM3uf+G). The Tamura and Nei substitution model, which was the closest available model in Arlequin, was used to calculate pairwise and global Φ_*ST*_ values, and to estimate genetic diversity at each site. Hierarchical Analyses of Molecular Variance (AMOVA) based on *F*_*ST*_ were carried out to partition the genetic variance across both space (ocean gyres) and time (sampling years), for both marker types. In these analyses, we tested for population structure under the following groupings: with samples stratified by (1) northern and southern subtropical gyres (2 gyres), and (2) across two sampling years (2010, 2012). Global *F*_*ST*_ values were estimated using non-hierarchical AMOVAs among all samples, as well as among subsets of the data across ocean gyres and sampling years. Significance was tested with 10,000 permutations of genotypes or haplotypes among populations. Principal coordinate analysis (PCA) plots of linearized pairwise *F*_*ST*_ values based on both mtCOII and microsatellite data were used to visualize spatial and temporal genetic differentiation among samples. Population structure was further examined using a Bayesian clustering method implemented in STRUCTURE [45,46] for microsatellite loci. We used admixture and correlated allele frequency models, with a burn-in of 10^5^ steps followed by 10^6^ steps, with and without using sampling location as a prior. We ran these analyses for each of the 2010 and 2012 datasets using *K* = 1 to *K* = 10, and for the dataset of combined years using *K* = 1 to *K* = 20. We ran three separate replicates for each K to investigate consistency of Pr(*X|K*). The true *K* was evaluated by visual inspection of barplots and comparing Pr(*X|K*) across *K* values.

## Results

### Genetic Diversity

A total of 58 mtCOII haplotypes occurred among the 472 *H*. *longicornis* sp. 1 animals sequenced, with an average of 7 haplotypes observed at each sampling site (S1 Table). Average nucleotide diversity across all samples was 0.00553, with a range from 0.00333–0.00774. No difference was observed in the number of haplotypes, haplotype diversity, or nucleotide diversity between samples collected in 2010 and 2012 (Mann-Whitney rank sum or t-tests, *P* >> 0.05 in all cases). However, there were significant differences between the northern and southern subtropical gyres in the number of mtCOII haplotypes (Mann-Whitney rank sum, *P* = 0.006), haplotype diversity (t-test, *P* < 0.001), and nucleotide diversity (Mann-Whitney rank sum, *P* = 0.021), with higher diversity observed in the southern gyre.

Our seven microsatellite markers were moderately polymorphic, with the average number of alleles per locus ranging from 4.9 to 6.9 across all samples (S2 Table). The average observed and expected heterozygosities across all loci ranged across 0.28–0.51 and 0.44–0.58, respectively. Only one of 148 locus-by-population comparisons showed deviation from HWE after Bonferroni correction. MICROCHECKER found 4 of 7 microsatellite markers exhibiting null alleles, with null allele frequency at these markers ranging from 0.12 to 0.34. However, as noted by Andrews et al. [28] for these same microsatellite markers, when pairwise and global *F*_*ST*_ values are calculated both with and without correction for null alleles, higher values are observed in the corrected *F*_*ST*_ values. For this reason, we consider it conservative to present these values as uncorrected for null alleles, as this is least likely to identify spurious, elevated structure among populations.

### Population structure

**Fig 2:**
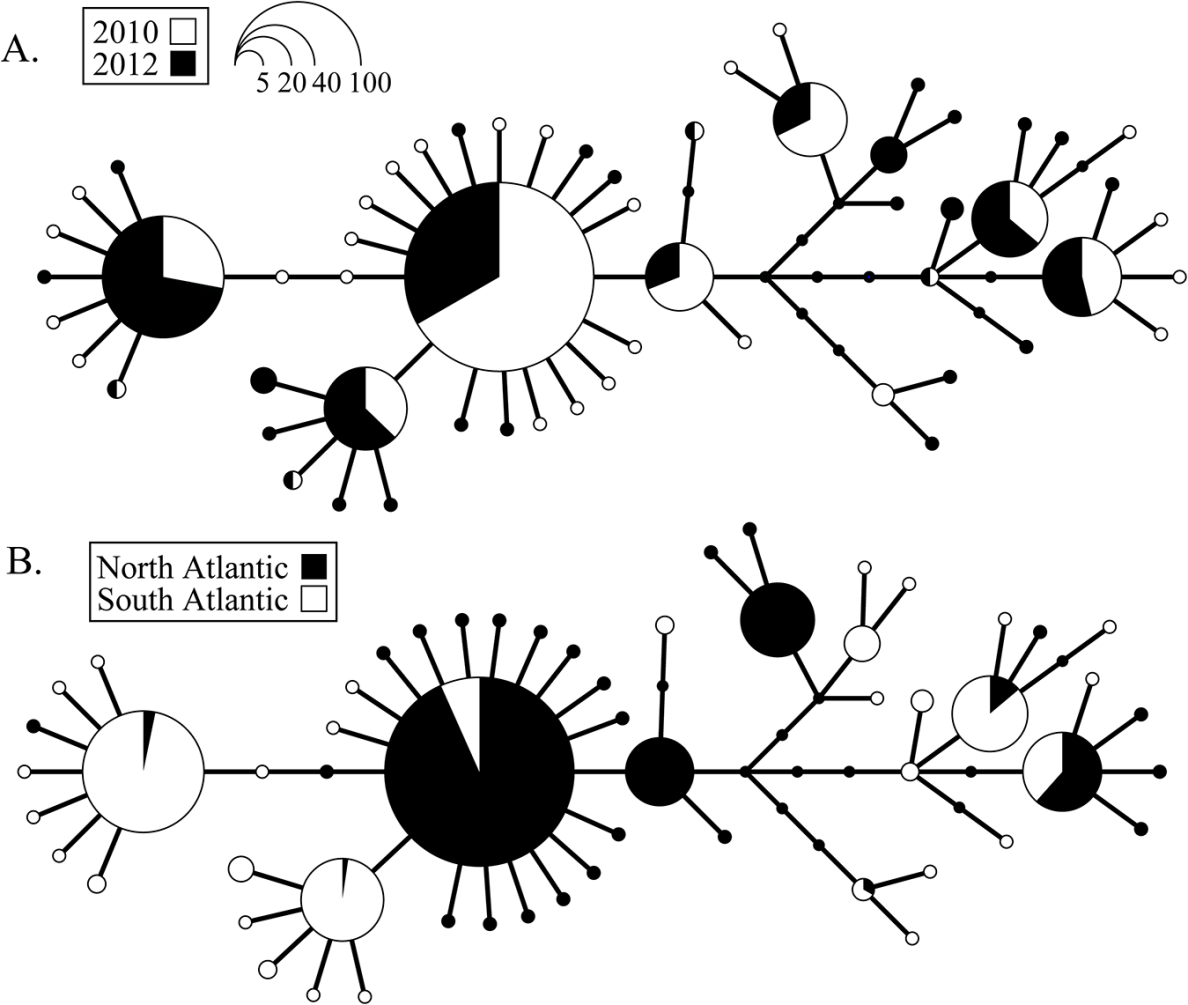
Haplotype genealogy of 58 mitochondrial cytochrome oxidase subunit II (mtCOII) haplotypes sampled in *Haloptilus longicornis* species 1. Genealogies are plotted to illustrate the extent of haplotype sharing across (A) sampling years 2010, 2012, and (B) ocean gyres (northern and southern subtropical gyres).

**Fig 3:**
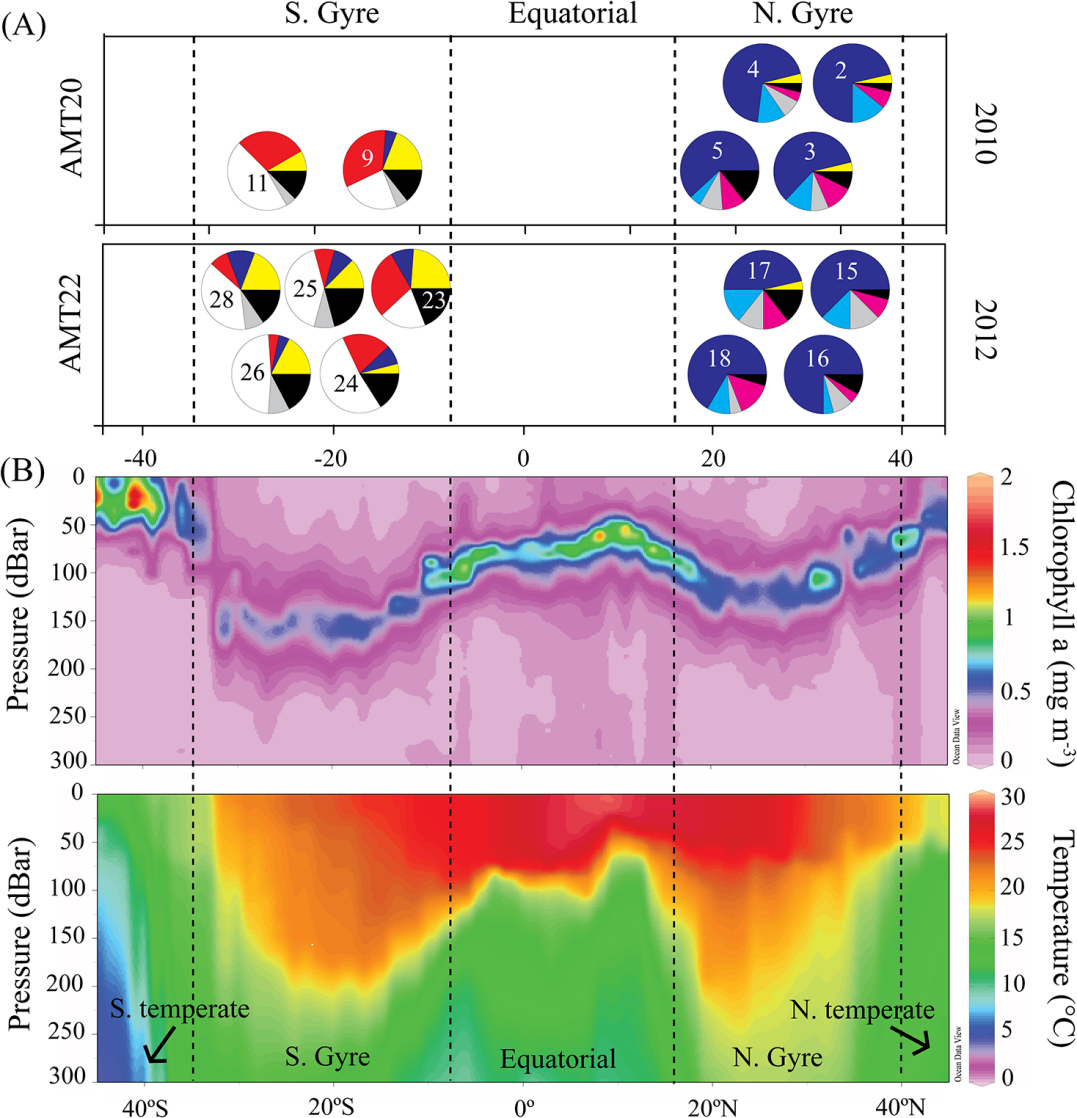
Temporal stability in the cytochrome oxidase II (mtCOII) haplotype composition of *Haloptilus longicornis* species 1 across ocean gyres in two years (2010, 2012). (A) mtCOII haplotype frequencies within each subtropical gyre, sampled in 2010 (top) and 2012 (bottom). Each pie represents a population sample, with PopID as listed in Table 1 and shown in Fig 1 (number inside the pie). Each color is a unique haplotype; black indicates all haplotypes occurring only once in the sample. Population samples with N > 20 were included. (B) Biogeographic provinces across the Atlantic Ocean shown in section plots of (top) Chlorophyll *a*, and (bottom) seawater temperature (0–300 m), from the 2012 AMT22 cruise.

**Fig 4:**
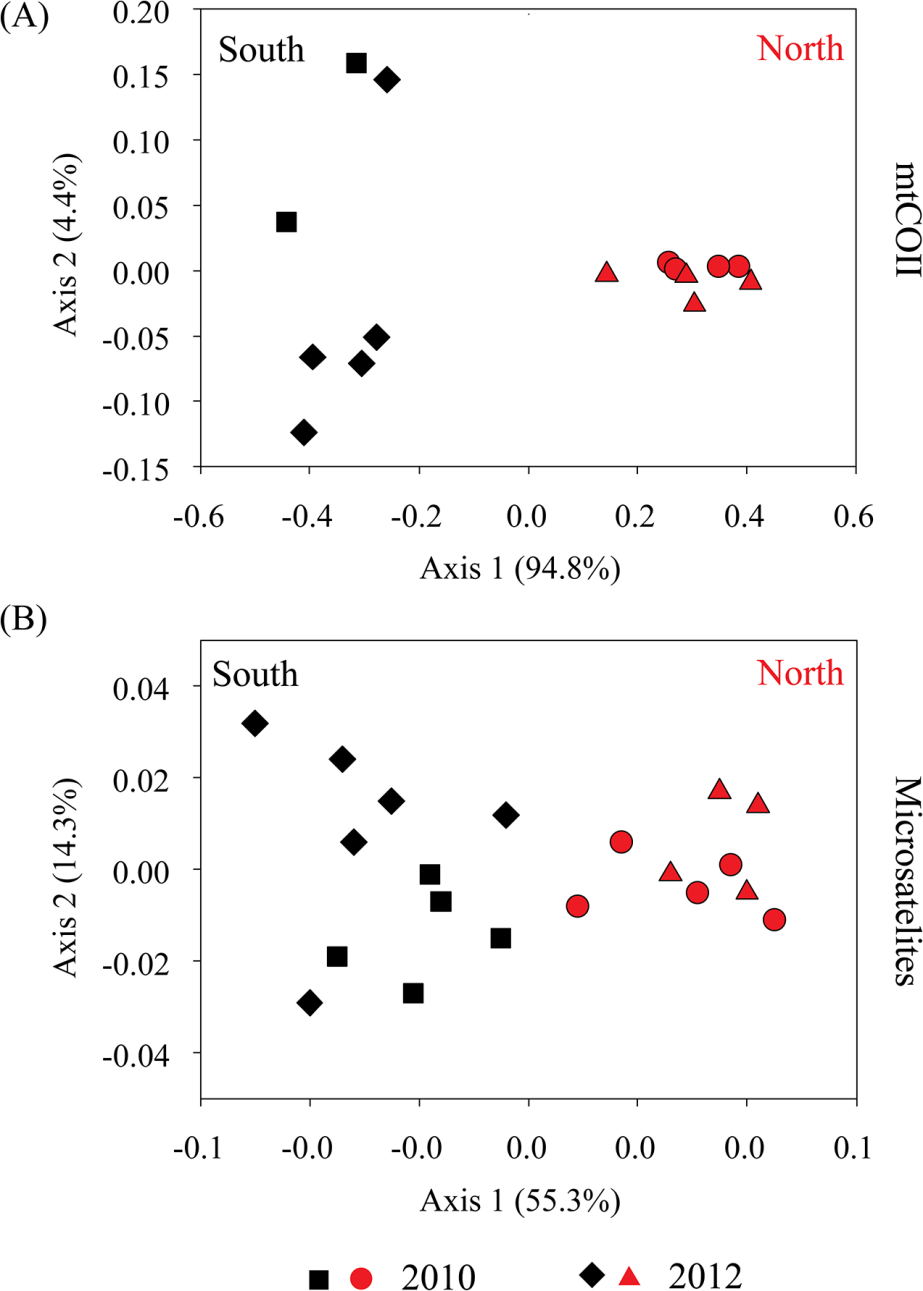
Spatial structure among *Haloptilus longicornis* sp. 1 subtropical gyre populations shown in principal coordinate analysis plots, based on linearized pairwise *F*_*ST*_ values among sampling sites. (A) Results from mtCOII sequence data, shown for 15 sampling sites in 2010 and 2012. (B) Results from 7 microsatellite loci, for 20 sampling sites in 2010 and 2012. In both plots, red indicates north gyre and black indicates south gyre sampling sites, and the circle and square symbols are from 2010 and the diamond and triangle symbols are from 2012. Note the absence of separation in temporal samples within each gyre.

There was significant genetic differentiation among samples at both mtCOII (global *F*_*ST*_ = 0.175, *P* < 0.0001, global Φ_*ST*_ = 0.048, *P* = 0.0001) and microsatellite markers (global *F*_*ST*_ = 0.01, *P* < 0.0001, Table 2). The dominant pattern in the data was strong spatial differentiation among populations in the northern and southern subtropical gyres. In hierarchical AMOVA analyses, significant amounts of genetic variation were partitioned among the two ocean gyres (*F*_*CT*_ = 0.285, *P* < 0.0001 for mtCOII, *F*_*CT*_ = 0.013, *P* < 0.0001 for microsatellites), while no significant results were found among the two sampling years (Table 2). These results held for analyses including all samples, as well as for analyses with samples collected only in either 2010 or 2012 and for samples collected only within each gyre across both years. Results of non-hierarchical AMOVA analyses of both mtCOII and microsatellite data also were significant when they included samples distributed across both ocean gyres, either including both years (global mtCOII *F*_*ST*_ = 0.175, *P* < 0.0001, global msat *F*_*ST*_ = 0.010, *P* < 0.0001), 2012 only (global msat *F*_*ST*_ = 0.013, *P* < 0.0001, global mtCOII *F*_*ST*_ = 0.163, *P* < 0.0001), or 2010 only for the mtCOII data (global mtCOII *F*_*ST*_ = 0.201, *P* < 0.0001; Table 2). All among-gyre pairwise *F*_*ST*_ values based on mtCOII were highly significant, irrespective of sampling year (*P* < 0.0001; S3 Table), with values ranging up to 0.407. The majority of among-gyre pairwise Φ_*ST*_ comparisons also were significant, although pairwise Φ_*ST*_ values were lower than *F*_*ST*_ values for the same sample comparisons (S3 Table). Among-gyre values also were more often significant in microsatellite pairwise *F*_*ST*_ (46 of 99 comparisons), than were within-gyre comparisons (5 of 91 comparisons significant; S4 Table). Haplotype genealogies illustrated broad mtCOII haplotype sharing among years (Fig 2A), but with spatial separation among northern and southern gyres (Fig 2B). However, there was no deep phylogenetic division between gyres, with several haplotypes that were restricted to the North Atlantic only one or a few mutational steps away from haplotypes that were unique to or shared with the South Atlantic. The dominant mtCOII haplotypes differed between the northern and southern subtropical gyres, with haplotype 3 (blue, Fig 3A) dominant in the northern gyre and haplotype 6 (white) dominant in the southern gyre (across both years; Fig 3A). Haplotype dominance within each gyre was stable across the two sampling years. Principal coordinate analysis (PCA) of linearized pairwise *F*_*ST*_ values among all samples separated populations primarily along an axis defined by space rather than time, with 94.8% (mtCOII) and 55.3% (msat) of the variation explained by the first principal coordinate (PCo1; Fig 4). For both mtCOII and microsatellite marker types, there was no notable separation of samples by sampling year (2010, 2012). Bayesian clustering analyses using microsatellites indicated *K* = 1 for all datasets (2010, 2012, and both years combined); however, this type of analysis lacks power for levels of divergence comparable to our microsatellite dataset [46]. In sum, at large spatial scales, population structure among northern and southern subtropical gyres is the dominant genetic pattern.

**Table 2:** Results of hierarchical and non-hierarchical AMOVA analyses for *Haloptilus longicornis* species 1.

**(A) Samples (grouping)**	**marker**	**N samples**	**N indiv.**	***F***_***CT***_	***P*-value**
all samples (2 gyres)	msat	20	840	**0.0130**	**< 0.0001**
all samples (2 years)	msat	20	840	-0.0007	0.6896
North gyre (2 years)	msat	9	394	0.0009	0.3491
South gyre (2 years)	msat	11	446	-0.0003	0.5462
2010 samples (2 gyres)	msat	10	367	**0.0100**	**0.0076**
2012 samples (2 gyres)	msat	10	473	**0.0150**	**0.0051**
all samples (2 gyres)	mtCOII	15	363	**0.2850**	**< 0.0001**
all samples (2 years)	mtCOII	15	363	-0.0060	0.4014
North gyre (2 years)	mtCOII	8	199	-0.0030	0.7992
South gyre (2 years)	mtCOII	7	164	0.0110	0.1414
2010 samples (2 gyres)	mtCOII	6	147	0.3440	0.0652
2012 samples (2 gyres)	mtCOII	9	216	**0.2550**	**0.0063**
**(B) Samples**	**marker**	**N samples**	**N indiv.**	**Global *F***_***ST***_	***P*-value**
all samples	msat	20	840	**0.010**	**< 0.0001**
North gyre (2 years)	msat	9	394	0.002	0.6761
South gyre (2 years)	msat	11	446	0.004	0.2638
2010 samples (10 locations)	msat	10	367	0.007	0.1158
2012 samples (10 locations)	msat	10	473	**0.013**	**< 0.0001**
all samples	mtCOII	15	363	**0.175**	**< 0.0001**
North gyre (2 years)	mtCOII	8	199	-0.015	0.9277
South gyre (2 years)	mtCOII	7	164	0.012	0.1371
2010 samples (6 locations)	mtCOII	6	147	**0.201**	**< 0.0001**
2012 samples (9 locations)	mtCOII	9	216	**0.163**	**< 0.0001**

(A) Hierarchical AMOVA with partitioning of genetic variance between gyres (North, South) and across sampling years (2010, 2012). (B) Global *F*_*ST*_ values (and p-values) for non-hierarchical AMOVA analyses. Results for both microsatellite (msat) and mitochondrial (mtCOII) datasets are reported. **Bold** indicates significance at *P* < 0.01. See Table 1 for a detailed listing of all samples, and the text for description of the groupings in A.

At the within-gyre scale, a small number of sample comparisons also were significant, suggesting chaotic genetic patchiness at fine geographic scales. One pairwise Φ_*ST*_ value based on mtCOII sequence data was significant in a within-gyre comparison (corrected for multiple comparisons, S3 Table). Pairwise *F*_*ST*_ values based on microsatellite data were significant at within-gyre scales in three cases, with two cases involving a comparison between years and one case involving a comparison among sites, both of which were sampled in 2012 (S4 Table). Sample sizes were high at these sites, with between 37 and 60 animals genotyped from these locations.

## Discussion

Although stochastic processes are well known to underlie temporal genetic change in many marine species [1,47,48], very few studies have examined temporal variation in the marine holoplankton. Because holoplanktonic species are similar to many other marine species in being characterized by large population size, high dispersal capability, and broad geographic range, we might also expect them to exhibit temporal stability in allele frequencies and weak population genetic structure due to the combined effects of low genetic drift and high migration among sites. However, given that the pelagic habitat and the planktonic animals it contains are in constant motion, there also is uncertainty regarding whether the genetic composition of plankton in any given ocean region would be stable over time. Results reported here are the first to document that genetically distinct populations of planktonic species persist over time within subtropical gyres, even though these populations are continuously undergoing advection and diffusion in the open ocean. Our results are particularly interesting because they appear counter-intuitive given the highly dispersive nature of the pelagic habitat.

Our results suggest that temporal genetic change in zooplankton populations is heavily influenced by the spatial scale of study. At large spatial scales, we observed temporal stability in the common mesopelagic copepod *Haloptilus longicornis* species 1. The strongest signal in our data is of genetic differentiation between northern and southern subtropical gyres, irrespective of sampling year. The significant and strong spatial sub-structuring observed among northern and southern gyres in this species in 2010 was also present in 2012, with the same haplotypes and alleles dominant in each gyre across the two years. Samples collected in 2012 were not consistently differentiated from those collected in the same ocean gyre in 2010, and there was no significant partitioning of genetic variation among years in this species (hierarchical AMOVA, Table 2). The two-year time span between samples likely corresponds to on the order of 25–30 generations for this species [49], given the seawater temperature in their core subtropical distribution, which should be sufficient to detect stochastic variation in the genetic composition of cohorts or generations. Similar results of an absence of differentiation among samples were found in two other studies examining the temporal component of population structure in marine zooplankton [25–27]. In contrast to *H*. *longicornis*, these species have temperate-boreal (*Meganyctiphanes norvegica*, *Sagitta setosa*) or circum-antarctic (*Euphausia superba*) distribution patterns and temporal stability was only examined for a subset of geographic samples. The shared observation of genetically distinct populations that persist through time in the open sea suggests that temporal stability in large-scale population structure may not be uncommon for holozooplankton in a variety of habitats. Our results support our initial expectations that plankton populations within subtropical gyres represent distinct gene pools that persist over time.

Our results, along with previous studies, also indicate that temporal stability of population genetic structure at large spatial scales is coupled with chaotic genetic patchiness, or weak and ephemeral genetic differentiation, at fine spatial scales. Marine zooplankton have highly patchy abundance and distribution at the submesoscale (1–10 km; [50,51]), and chaotic genetic patchiness may occur on spatial scales of meters to kilometers, or temporal scales of days to months. Statistical heterogeneity in the genetic composition of zooplankton samples on the mesoscale and submesoscale has been observed in a number of prior studies, although the interpretation of this pattern has been varied. Bortolotto et al. [26] included temporal replicates of krill samples from the Ross Sea (5 years) and South Georgia (2 years) and concluded that although there was a single panmictic population of krill on a large geographic scale (Southern Ocean), there was also evidence for subtle, though not statistically significant, spatial and temporal heterogeneity. Other examples include spatial genetic patchiness in the copepod *Metridia pacifica* populations across mesoscale coastal upwelling filaments and eddies in the California Current [52,53], as well as outlier samples that show high pairwise *F*_*ST*_ values unrelated to geographic distance or ocean habitat among sites in several other studies (*Eucalanus hyalinus*, *Pleuromamma xiphias*, [20,21]). A number of other studies report unexplained genetic heterogeneity at various spatial or temporal scales in zooplankton species (e.g., [54–59]); however, in some cases, these observations were confounded by sampling artifacts and low sample size (spurious, significant *F*_*ST*_). Genetic patchiness also can be seen in the data reported here for *Haloptilus longicornis*. There are population samples within both the northern and southern gyres that show weak but significant differentiation relative to other samples within the same gyre (both marker types, S3 & S4 Tables). As in other marine species, this patchiness can include either a temporal or spatial component (or both; S3 & S4 Tables). There is little understanding of the mechanisms that underlie this small-scale genetic patchiness in marine zooplankton, but in addition to variance in reproductive success (and clonal reproduction; [60]), they may include biological or bio-physical aggregation of animals (e.g., swarms, thin-layers, vertical migration; [61,62]), and selective mortality across the life history. In sum, at smaller spatial scales, zooplankton may exhibit genetic patchiness in either temporal or spatial domains, as is well-described in many other highly-dispersing marine species (e.g., [10,17,18,63–65]).

Operationally, our results suggest that the broad-scale genetic patterns of population structure within holozooplankton species can be captured with limited temporal sampling given sufficient sampling intensity (sample size). This result is reassuring, as the majority of prior studies have used samples from a wide range of sampling dates, and interpreted the patterns observed exclusively in a spatial context (e.g., [20,21,28,30,66–71]). Given spatially extensive, in many cases circumglobal, range distributions for marine zooplankton, this approach has been necessary to collecting material across the distributional range of the species. However, it has always been recognized that the lack of temporally-stratified sampling resulted in poor understanding of the extent to which our interpretation of population structure in marine holoplankton was confounded by genetic variation across both space and time (e.g., [25–27,72]). Results reported here are therefore important in documenting that genetic structure occurring over large spatial scales is temporally persistent (Table 2). If this is true for other species, then combining temporally-stratified samples to examine spatial structure is unlikely to impede interpretation of the larger-scale population structure in these species.

In summary, this study is the first to demonstrate that both the genetic composition of plankton populations within oceanic gyres and the spatial patterns of population sub-structure across ocean gyres are persistent through time. This large-scale pattern of significant and temporally-stable genetic structure for marine zooplankton is coupled with chaotic genetic patchiness at finer spatial scales (in this case, within subtropical gyres). Our results demonstrate temporal stability in the genetic structure of a ‘high dispersal’ marine species, and are particularly important given the dispersive nature of the pelagic habitat and very high vagility of planktonic species. Additional work is needed to understand the extent and underlying causes of fine-scale genetic patchiness in the plankton, as well as to interpret its biological impact on species. Confirmation of the observations of temporally stable genetic structure over large spatial scales from other zooplankton species also would lend greater support to the conclusion that these patterns are the norm for the holozooplankton.

## Supporting Information

S1 File Microsatellite genotypes for all specimens, with sample ID and cruise and station as listed for each specimen. Please contact the corresponding author regarding subsequent use of these data (egoetze@hawaii.edu).
(CSV)

S1 Table Mitochondrial cytochrome oxidase subunit II (mtCOII) summary statistics and diversity indices for population samples, including only specimens of *Haloptilus longicornis* species 1. Collection sites for plankton samples included in this study, from (A) the 2010 AMT cruise (AMT20), and (B) the 2012 AMT cruise (AMT22). Pop ID = the population identifier referred to throughout the manuscript; Station = the cruise and station number of each sample; N = the number of adult females included; H = the number of haplotypes; *h* = haplotype diversity; *π* = nucleotide diversity.
(PDF)

S2 Table Microsatellite diversity indices for population samples, including only specimens of *Haloptilus longicornis* species 1. Collection sites for plankton samples included in this study, from (A) the 2010 AMT cruise (AMT20), and (B) the 2012 AMT cruise (AMT22). Pop ID = the population identifier referred to throughout the manuscript; Station = the cruise and station number of each sample; N = the number of adult females included. *H*_*o*_ and *H*_*e*_ are the observed and expected heterozygosity averaged across all microsatellite loci for each population. *k* = average number of alleles per locus.
(PDF)

S3 Table Pairwise *F*_*ST*_ (below diagonal) and Φ_ST_ (above diagonal) values between sample sites in the Atlantic Ocean for *Haloptilus longicornis* sp 1, based on mtCOII data. Samples are grouped by ocean gyres, with sample site numbers as in Tables 1 & 2. Material from both AMT20 (2010) and AMT22 (2012) are included. Significant values (*P* < 0.05) are shaded grey, **bold** indicates significance following correction for multiple comparisons (FDR).
(PDF)

S4 Table Pairwise *F*_*ST*_ values between sample sites in the Atlantic Ocean for *Haloptilus longicornis* sp 1, based on microsatellite data. Samples are grouped by ocean gyres, with sample site numbers as in Tables 1 & 2. Material from both AMT20 (2010) and AMT22 (2012) are included. Significant values (P < 0.05) are shaded grey, **bold** indicates significance following correction for multiple comparisons (FDR).
(PDF)
