# Supplementary material for: Temporal Stability of Genetic Structure in a Mesopelagic Copepod
Source: PLoS One. 2015 Aug 24;10(8):e0136087. doi: 10.1371/journal.pone.0136087 (PMC4547763; doi:10.1371/journal.pone.0136087)
Supplement: S1 Table — Collection sites for plankton samples included in this study, from (A) the 2010 AMT cruise (AMT20), and (B) the 2012 AMT cruise (AMT22). Pop ID = the population identifier referred to throughout the manuscript; Station = the cruise and station number of each sample; N = the number of adult females included; H = the number of haplotypes; h = haplotype diversity; π = nucleotide diversity. (PDF) [file pone.0136087.s002.pdf]

Table S1. Mitochondrial cytochrome oxidase subunit II (mtCOII) summary statistics and diversity indices for population samples, including only specimens of *Haloptilus longicornis* species 1. Collection sites for plankton samples included in this study, from (A) the 2010 AMT cruise (AMT20), and (B) the 2012 AMT cruise (AMT22). Pop ID = the population identifier referred to throughout the manuscript; Station = the cruise and station number of each sample; N = the number of adult females included; H = the number of haplotypes; *h* = haplotype diversity; π = nucleotide diversity.

**Pop ID**	**Station**	**N**	**H**	***h***	**π**
(A) 2010 Cruise, Atlantic Meridional Transect Cruise 20					
2	AMT20-09	28	5	0.479	0.0033
3	AMT20-11	27	7	0.638	0.0047
4	AMT20-13	26	6	0.517	0.0051
5	AMT20-15	21	7	0.619	0.0048
9	AMT20-24	21	8	0.824	0.0072
10	AMT20-25	24	7	0.721	0.0049
(B) 2012 Cruise, Atlantic Meridional Transect Cruise 22					
15	AMT22-15	24	5	0.594	0.0056
16	AMT22-21	23	6	0.459	0.0033
17	AMT22-25	27	8	0.744	0.0059
18	AMT22-29	21	5	0.548	0.0033
23	AMT22-49	21	8	0.848	0.0076
24	AMT22-51	24	7	0.678	0.0045
25	AMT22-55	24	10	0.815	0.0077
26	AMT22-57	23	8	0.751	0.0072
28	AMT22-60	27	9	0.826	0.0077
