# Supplementary material for: Temporal Stability of Genetic Structure in a Mesopelagic Copepod
Source: PLoS One. 2015 Aug 24;10(8):e0136087. doi: 10.1371/journal.pone.0136087 (PMC4547763; doi:10.1371/journal.pone.0136087)
Supplement: S2 Table — Collection sites for plankton samples included in this study, from (A) the 2010 AMT cruise (AMT20), and (B) the 2012 AMT cruise (AMT22). Pop ID = the population identifier referred to throughout the manuscript; Station = the cruise and station number of each sample; N = the number of adult females included. H o and H e are the observed and expected heterozygosity averaged across all microsatellite loci for each population. k = average number of alleles per locus. (PDF) [file pone.0136087.s003.pdf]

Table S2. Microsatellite diversity indices for population samples, including only specimens of *Haloptilus longicornis* species 1. Collection sites for plankton samples included in this study, from (A) the 2010 AMT cruise (AMT20), and (B) the 2012 AMT cruise (AMT22). Pop ID = the population identifier referred to throughout the manuscript; Station = the cruise and station number of each sample; N = the number of adult females included. $H_o$ and $H_e$ are the observed and expected heterozygosity averaged across all microsatellite loci for each population. $k$ = average number of alleles per locus.

| **Pop ID** | **Station** | **N** | $H_E$ | $H_O$ | ***k*** | ***Allele richness*** |
|---|---|---|---|---|---|---|
| *(A) 2010 Cruise, Atlantic Meridional Transect Cruise 20* | | | | | | |
| 2 | AMT 20-09 | 37 | 0.500 | 0.318 | 4.86 | 4.55 |
| 3 | AMT 20-11 | 43 | 0.480 | 0.362 | 4.86 | 4.26 |
| 4 | AMT 20-13 | 45 | 0.492 | 0.367 | 5.43 | 4.66 |
| 5 | AMT 20-15 | 37 | 0.478 | 0.342 | 5.14 | 4.62 |
| 6 | AMT 20-16 | 30 | 0.584 | 0.508 | 4.86 | 4.75 |
| 9 | AMT 20-24 | 37 | 0.527 | 0.425 | 6.86 | 6.00 |
| 10 | AMT 20-25 | 41 | 0.488 | 0.383 | 5.43 | 4.84 |
| 11 | AMT 20-26 | 37 | 0.501 | 0.340 | 5.86 | 5.17 |
| 12 | AMT 20-27 | 32 | 0.487 | 0.282 | 5.71 | 5.37 |
| 13 | AMT 20-28 | 28 | 0.494 | 0.349 | 5.14 | 5.04 |
| | **Total** | **367** | | | | |
| *(B) 2012 Cruise, Atlantic Meridional Transect Cruise 22* | | | | | | |
| 15 | AMT22-15 | 47 | 0.525 | 0.439 | 6.00 | 5.10 |
| 16 | AMT22-21 | 60 | 0.527 | 0.385 | 6.14 | 5.04 |
| 17 | AMT22-25 | 45 | 0.488 | 0.330 | 5.00 | 4.29 |
| 18 | AMT22-29 | 50 | 0.479 | 0.359 | 5.43 | 4.39 |
| 23 | AMT22-49 | 51 | 0.485 | 0.358 | 5.71 | 4.76 |
| 24 | AMT22-51 | 40 | 0.443 | 0.378 | 5.71 | 4.80 |
| 25 | AMT22-55 | 52 | 0.491 | 0.414 | 6.00 | 4.79 |
| 26 | AMT22-57 | 40 | 0.512 | 0.424 | 5.71 | 5.09 |
| 27 | AMT22-58 | 42 | 0.508 | 0.440 | 5.43 | 4.71 |
| 28 | AMT22-60 | 46 | 0.481 | 0.371 | 6.14 | 5.03 |
| | **Total** | **473** | | | | |
