# Supplementary material for: Temporal Stability of Genetic Structure in a Mesopelagic Copepod
Source: PLoS One. 2015 Aug 24;10(8):e0136087. doi: 10.1371/journal.pone.0136087 (PMC4547763; doi:10.1371/journal.pone.0136087)
Supplement: S3 Table — Samples are grouped by ocean gyres, with sample site numbers as in Tables 1 & 2. Material from both AMT20 (2010) and AMT22 (2012) are included. Significant values (P < 0.05) are shaded grey, bold indicates significance following correction for multiple comparisons (FDR). (PDF) [file pone.0136087.s004.pdf]

Supplemental Table 3. Pairwise F_{ST} (below diagonal) and Φ_{ST} (above diagonal) values between sample sites in the Atlantic Ocean for *Haloptilus longicornis* sp 1, based on mtCOII data. Samples are grouped by ocean gyres, with sample site numbers as in Tables 1 & 2. Material from both AMT20 (2010) and AMT22 (2012) are included. Significant values ($P < 0.05$) are shaded grey, **bold** indicates significance following correction for multiple comparisons (FDR).

		North Atlantic Gyre								South Atlantic Gyre						
		2	3	4	5	15	16	17	18	9	11	23	24	25	26	28
North Atlantic Gyre	2	****	-0.016	-0.015	0.011	0.009	-0.002	0.006	-0.032	**0.132**	**0.133**	**0.136**	**0.132**	**0.129**	**0.171**	**0.104**
	3	-0.012	****	-0.035	-0.028	-0.031	-0.015	-0.028	-0.030	**0.070**	**0.078**	**0.078**	**0.109**	0.065	**0.140**	0.056
	4	-0.027	-0.021	****	-0.027	-0.033	-0.014	-0.029	-0.026	0.064	0.072	**0.073**	**0.107**	0.058	**0.136**	0.049
	5	-0.007	-0.030	-0.023	****	-0.029	-0.027	-0.019	-0.019	0.061	0.070	0.057	**0.102**	0.055	**0.127**	0.044
	15	-0.012	-0.032	-0.029	-0.033	****	0.004	-0.038	-0.007	0.041	0.055	0.056	**0.117**	0.036	**0.142**	0.043
	16	-0.013	-0.005	-0.027	-0.020	-0.016	****	0.013	-0.027	**0.110**	**0.112**	**0.096**	**0.108**	**0.105**	**0.138**	0.073
	17	0.023	-0.022	0.008	-0.012	-0.018	0.033	****	-0.008	0.043	0.053	0.059	**0.108**	0.042	**0.135**	0.047
	18	-0.026	-0.032	-0.027	-0.033	-0.031	-0.021	-0.007	****	**0.103**	**0.100**	**0.098**	**0.096**	**0.104**	**0.134**	0.075
South Atlantic Gyre	9	**0.331**	**0.243**	**0.304**	**0.253**	**0.267**	**0.336**	**0.182**	**0.282**	****	-0.027	-0.022	0.074	-0.025	0.072	-0.007
	11	**0.403**	**0.317**	**0.379**	**0.326**	**0.339**	**0.407**	**0.249**	**0.348**	0.005	****	-0.033	0.028	0.000	0.029	-0.005
	23	**0.292**	**0.206**	**0.266**	**0.217**	**0.231**	**0.298**	**0.149**	**0.246**	-0.035	0.029	****	0.027	-0.010	0.019	-0.021
	24	**0.388**	**0.305**	**0.364**	**0.313**	**0.325**	**0.391**	**0.238**	**0.330**	0.048	-0.020	0.066	****	**0.094**	-0.031	0.029
	25	**0.314**	**0.228**	**0.287**	**0.235**	**0.248**	**0.314**	**0.163**	**0.257**	0.026	0.004	0.028	-0.007	****	0.084	-0.029
	26	**0.367**	**0.279**	**0.340**	**0.289**	**0.301**	**0.370**	**0.210**	**0.310**	0.054	0.019	0.052	-0.001	-0.027	****	0.020
	28	**0.287**	**0.206**	**0.261**	**0.214**	**0.227**	**0.289**	**0.145**	**0.235**	0.021	0.021	0.009	0.013	-0.025	-0.020	****
