# Supplementary material for: Temporal Stability of Genetic Structure in a Mesopelagic Copepod
Source: PLoS One. 2015 Aug 24;10(8):e0136087. doi: 10.1371/journal.pone.0136087 (PMC4547763; doi:10.1371/journal.pone.0136087)
Supplement: S4 Table — Samples are grouped by ocean gyres, with sample site numbers as in Tables 1 & 2. Material from both AMT20 (2010) and AMT22 (2012) are included. Significant values (P < 0.05) are shaded grey, bold indicates significance following correction for multiple comparisons (FDR). (PDF) [file pone.0136087.s005.pdf]

Supplementary Table 4. Pairwise F_{ST} values between sample sites in the Atlantic Ocean for *Haloptilus longicornis* sp 1, based on microsatellite data. Samples are grouped by ocean gyres, with sample site numbers as in Tables 1 & 2. Material from both AMT20 (2010) and AMT22 (2012) are included. Significant values (P < 0.05) are shaded grey, **bold** indicates significance following correction for multiple comparisons (FDR).

		North Atlantic Gyre									South Atlantic Gyre										
		2	3	4	5	6	15	16	17	18	9	10	11	12	13	23	24	25	26	27	28
North Atlantic Gyre	2	****																			
	3	-0.003	****																		
	4	-0.003	-0.006	****																	
	5	0.003	0.001	0.010	****																
	6	-0.008	-0.013	-0.003	0.003	****															
	15	-0.013	-0.001	-0.004	-0.002	-0.002	****														
	16	-0.001	-0.005	0.006	0.001	-0.002	0.000	****													
	17	0.007	0.009	**0.014**	0.004	0.001	-0.003	**0.014**	****												
	18	0.002	0.000	0.002	0.010	0.004	-0.004	0.010	0.004	****											
South Atlantic Gyre	9	-0.005	0.013	-0.002	0.013	0.013	0.004	**0.014**	**0.022**	0.011	****										
	10	0.009	0.009	0.004	**0.022**	0.005	0.010	**0.017**	**0.021**	**0.017**	0.007	****									
	11	0.006	0.014	0.007	**0.021**	0.013	**0.012**	**0.019**	**0.028**	**0.023**	0.005	0.001	****								
	12	0.009	**0.020**	0.010	**0.025**	0.015	0.010	**0.022**	**0.030**	**0.028**	0.001	0.001	-0.005	****							
	13	0.001	0.002	-0.005	0.007	-0.006	-0.004	0.004	**0.014**	0.009	-0.004	-0.004	-0.005	-0.006	****						
	23	0.006	0.016	0.009	0.018	0.017	**0.017**	**0.022**	**0.025**	**0.026**	0.008	0.004	0.014	0.006	0.003	****					
	24	0.018	**0.030**	0.004	**0.032**	0.016	0.012	**0.035**	**0.037**	**0.032**	-0.001	0.006	0.002	-0.004	0.000	0.008	****				
	25	0.006	**0.021**	0.011	**0.023**	0.016	0.005	**0.026**	**0.021**	0.017	-0.006	0.008	0.000	-0.005	0.002	0.002	0.002	****			
	26	**0.018**	**0.034**	**0.018**	**0.051**	**0.032**	**0.024**	**0.039**	**0.044**	**0.030**	0.010	0.009	**0.019**	0.009	0.014	0.010	0.017	0.004	****		
	27	-0.002	0.004	0.004	0.013	-0.002	-0.002	0.005	0.011	0.009	0.001	0.000	0.005	0.001	-0.004	0.004	0.014	-0.001	0.008	****	
	28	0.008	**0.016**	0.012	**0.031**	0.015	**0.018**	**0.029**	**0.028**	**0.025**	0.008	0.003	0.010	0.005	0.003	-0.004	0.011	-0.002	0.002	0.000	****
